# HACR-Net: An Efficient hybrid attention network for MRI image super-resolution

**DOI:** 10.1371/journal.pone.0345637

**Published:** 2026-04-08

**Authors:** Abdulhamid Muhammad, Amir Hajian, Titipat Achakulvisut, Supavadee Aramvith

**Affiliations:** 1 Department of Electrical Engineering, Faculty of Engineering, Chulalongkorn University, Bangkok, Thailand; 2 Department of Biomedical Engineering, Faculty of Engineering, Mahidol University, Salaya, Nakhon Pathom, Thailand; 3 Multimedia Data Analytics and Processing Research Unit, Department of Electrical Engineering, Faculty of Engineering, Chulalongkorn University, Bangkok, Thailand; Jaypee University of Information Technology, INDIA

## Abstract

High-resolution Magnetic Resonance Imaging (MRI) plays an important role in clinical diagnosis and pathological assessment, due to its non-invasive nature and lack of ionizing radiation. However, the acquisition of high-resolution MRI is often constrained by hardware limitations and a prolonged scanning duration. To address these limitations, super-resolution (SR) techniques have been introduced to reconstruct high-resolution images from low-resolution inputs. However, despite these advances, existing methods often struggle to effectively extract shallow features, model complex contextual dependencies, and preserve fine anatomical details. To address these limitations, we propose a Hybrid Attention and Channel Retention Network (HACR-Net) for MRI image SR. HACR-Net incorporates a Hybrid Attention Module (HAM) to mitigate information loss during shallow feature extraction by jointly leveraging channel and spatial attention, enhancing informative features, and preserving spatially significant regions. A Multiscale Feature Aggregation Block (MFAB) is incorporated to capture global structural details, local texture, and high-frequency details. Complementing MFAB, the Channel Retention Attention Block (CRAB) enhances the recovery of fine contextual detail through a bottleneck design crafted to maintain a wider channel width and reduce information loss during feature compression. Extensive experiments on two benchmark datasets, IXI and BraTS2018, demonstrate that HACR-Net achieves high-performance reconstruction with only 1.67M parameters and 81.3G FLOPs, offering significant reductions in model size and computational cost compared to existing methods.

## Introduction

Medical imaging plays an important role in modern healthcare, facilitating early disease detection, precise treatment planning, and ongoing monitoring of various conditions. Commonly used modalities such as ultrasound, Positron Emission Tomography (PET), Computed Tomography (CT), and Optical Coherence Tomography (OCT) provide important diagnostic information [1–3]. However, each modality involves inherent trade-offs between spatial resolution, penetration depth, patient safety, and radiation exposure [4–6]. High-resolution (HR) Magnetic Resonance Imaging (MRI) offers a non-invasive and radiation-free alternative, making it valuable for clinical evaluation and pathological analysis [1,2,7]. However, acquiring HR MRI images remains challenging due to hardware limitations, prolonged acquisition times, the need for a sufficient signal-to-noise ratio (SNR), and patient discomfort during scanning [8–10]. These persistent limitations underscore the urgent need for strategies that can improve MRI image quality without further burdening patients or hardware systems. While this improvement could be pursued through costly equipment upgrades or extended scanning durations, both options present practical and economic constraints. A cost-effective alternative is the use of SR techniques, which reconstruct HR images from LR inputs [11,12]. SR offers a clinically viable solution and has become a central focus in medical image enhancement research [13].

Image SR is an ill-posed inverse problem, which does not have a single definite solution to generate accurate and high perceptual fidelity super-resolved images [14,15]. Recently, there has been substantial growth in deep learning-based models, driven by the powerful representational capabilities of convolutional neural networks (CNNs) and their efficient implementation for both forward and backward computations [16,17]. Dong et al. [18] were the first to introduce CNNs for the image SR task through their Super-Resolution Convolutional Neural Network (SRCNN), which consists of only three convolutional layers. Kim et al. increased the depth of the network using residual learning in VDSR [19]. However, SRCNN is constrained by its shallow depth, leading to poor reconstruction of fine textures and high-frequency details. In contrast VDSR [19] experiences training instability and model degradation with increasing network depth. Advanced strategies, such as residual learning and dense connections, are employed to address this limitation.

Zhang et al. [20] improved SR performance by proposing a Dense Residual Network (RDN), which effectively utilizes the hierarchical characteristics of a deeper network architecture. Similarly, Li et al. [21] introduced a multiscale residual network, designed to leverage features across multiple scales, thus mitigating the loss of image details. These approaches emphasize the importance of leveraging hierarchical and multiscale features for enhanced image reconstruction quality. Feng et al. [22] implemented a multistage aggregation network for SR reconstruction for multi-contrast MRI images. Still, this strategy might require extensive datasets and perform poorly with single-contrast images. Meanwhile, Weng et al. [23] developed a high-frequency-focused network designed to selectively enhance high-frequency details, which could potentially overlook low-frequency information that is essential for maintaining overall image coherence.

CNN-based SR methods rely on standard convolutional operations that treat all spatial locations and feature channels uniformly, thereby overlooking the varying diagnostic importance of different anatomical regions. Because convolutions focus primarily on local neighborhoods, these models struggle to capture long-range contextual dependencies which are essential for accurate medical image reconstruction. As a result, their early feature representations often lack sufficient emphasis on critical structures such as tissue boundaries, interfaces, and fine-grained pathological regions, ultimately limiting reconstruction quality [24,25]. As a result, they prioritize local feature extraction while neglecting the global spatial correlations necessary for reconstructing clinically relevant details. This shortcoming, is particularly evident in regions with complex tissue morphology. In addition, most of these methods treat all spatial pixels equally, an issue that conflicts with the nature of MRI data, where complex anatomical structures, diverse textures, and extensive background regions require adaptive spatial attention.

The spatial position of tissue textures is highly correlated with their complexities, so treating all pixels equally narrows the network’s ability to identify the most important regions for accurate reconstruction [26]. Hongbi et al. [27] introduced MFER to restore degraded high-resolution details through multi-level feature extraction and reconstruction modules. It map features from each level to the high-resolution space using deconvolution layers. Although MFER performs well in capturing global information, its local information recovery capabilities remain limited. Transformer models, with their self-attention mechanisms, capture long-range dependencies that are often overlooked by CNNs; however, they can be less effective in preserving the fine-grained textural details essential for accurate reconstruction. Their ability to retain fine-grained local features depends heavily on architectural design, and some variants may not achieve an optimal balance between global context and local detail [28,29].

While SR techniques have shown promising quantitative gains in MRI, their adoption in routine clinical practice remains limited [30]. Many deep learning–based SR methods prioritize pixel-wise accuracy or perceptual quality, often at the expense of preserving fine anatomical details and tissue contrasts that are essential for clinical interpretation [31]. Additionally, the high computational complexity and multi-stage inference pipelines of many state-of-the-art methods hinder real-time processing and seamless integration into clinical workflows [32]. These limitations highlight the need for SR approaches that balance reconstruction accuracy, structural fidelity, and computational efficiency for clinical use.

Existing traditional methods [18–20] employ simple, shallow feature extraction techniques, which are insufficient to capture the complex hierarchical relationships inherent in LR brain MRI images. Moreover, the lack of effective multi-feature aggregation constrains the network’s ability to preserve global anatomical coherence, such as the organization of ventricles and major white matter tracts, as well as fine-grained details. As a result, reconstructed images often face an inherent trade-off: sacrificing global structural integrity for local detail preservation, or losing fine textures in favor of broader contextual accuracy. Additionally, traditional SR architectures [33,34] also experience progressive information degradation in their processing pipelines, where fine-grained features are lost during successive feature transformation stages. The conventional approach of using narrow channel widths and aggressive dimensionality reduction results in the loss of minute but clinically significant details such as small vessel structures, cortical folding patterns, and early-stage pathological changes [27,35,36]. This information loss is compounded by the lack of effective feature retention mechanisms that can preserve and recover fine contextual information throughout the network’s forward pass.

**Fig 1:**
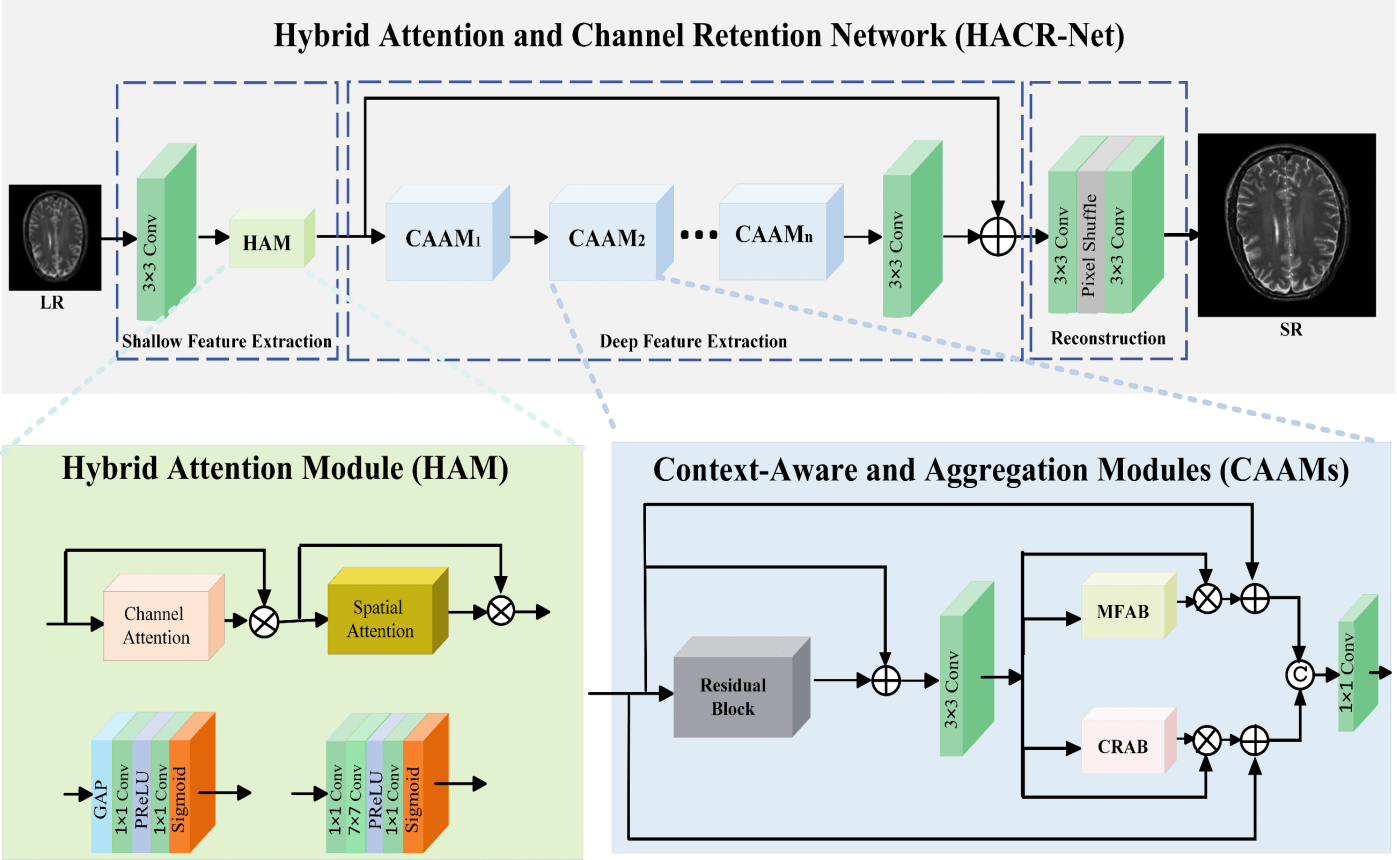
Hybrid Attention and Channel Retention Network (HACR-Net) Architecture.

To address these limitations, we propose Hybrid Attention and Channel Retention Network (HACR-Net) for MRI image SR. As illustrated in Fig 1, our framework integrates a Hybrid Attention Module that combines channel and spatial attention, enabling comprehensive and hierarchical extraction of shallow features. A robust multi-feature aggregation is achieved via parallel convolutions with varying kernel sizes, followed by adaptive channel refinement to model inter-channel dependencies and generate a globally consistent representation. In addition, the channel retention attention block mitigates progressive information degradation by maintaining a wider channel width and employing a bottleneck design to prevent information loss after feature reduction.

The main contributions of this research work are summarized as follows.

1. We propose a Hybrid Attention Module (HAM) that integrates channel and spatial attention to emphasize the most informative feature maps and spatially significant regions. This integration enables a comprehensive and hierarchical extraction of shallow features.
2. We design a Multi-Feature Aggregation Block (MFAB) to extract and integrate features across diverse receptive fields, enabling the simultaneous capture of fine-grained details and high-frequency information, thereby enhancing reconstruction fidelity across multiple scales in MRI data.
3. We propose a Channel-Retention Attention Block (CRAB) to enhance the recovery of fine contextual details. It employs a bottleneck design that maintains a broader channel width, thereby mitigating information loss during feature compression.
4. Extensive evaluations on the IXI and BraTS 2018 datasets demonstrate that our proposed HACR-Net outperforms the state-of-the-art methods in both quantitative and qualitative evaluations.

The remainder of this paper is organized as follows. The Related Work section reviews existing studies relevant to this research. The Methodology section describes the proposed method and its architecture. The Experiments section provides a comprehensive evaluation of the proposed approach, including datasets, implementation details, evaluation metrics, results and discussion, complexity analysis, and ablation studies. Finally, the Conclusion summarizes the main findings, and Future Work outlines potential directions for further research.

## Related work

Over the past decade, the field of computer vision has experienced substantial growth, with a diverse range of techniques being developed. Notably, the debut of the SRCNN [18] framework catalyzed numerous transformative advances in SR research. Subsequent research has focused on enhancing the architecture and training approaches of CNNs to improve their performance. For example, Kim et al. [19] introduced the VDSR model, employing deeper CNN layers and residual learning to enhance SR. Zhang et al. [20] developed a Residual Dense Network (RDN) that uses residual dense blocks to capture local features for high SR image reconstruction. However, the RDN overlooks fine details between channels and pixels.

Tai et al. later proposed the DRCN [37] and DRRN [38], which use recursive learning to achieve greater depth and efficiency with fewer parameters. However, their reliance on recursive structures can lead to challenges in training stability and require careful parameter initialization to avoid vanishing gradients. To address this, Li et al. [21] proposed a Multiscale Residual Network (MSRN) to extract fine features. At the same time, AWSRN [39] applies adaptive weighted learning to balance performance and computational demands for lightweight image SR efficiently. However, they rely on many scale rate calculations and focus on feature-level attention. Dai et al. [40] replaced global average pooling with second-order statistics to capture channel relationships but overlooked texture reconstruction. To address this, Tian et al. [41] introduced a coarse-to-fine convolutional neural network (CNN) that aggregates complementary information to stabilize the training process.

Fang et al. [42] developed a soft-edge-assisted network that enhances edge textures, thereby improving reconstruction of fine detail. Sun et al. [43] proposed a weighted multiscale residual network to strengthen textural detail recovery and preserve high-frequency information. However, these models exhibit insufficiently diverse feature-context modeling, which constrains their reconstruction abilities. Sun et al. [44] employed a large depth-wise convolution to enhance fine-grained details. However, these approaches struggle with effective learning and fail to address the relationships between feature-level and channel-level attention characteristics.

Attention mechanisms [45] are pivotal in improving feature representation in Image SR models by enhancing focus on key image features. Meanwhile, recent advancements in neural networks underscore the importance of capturing spatial correlations, suggesting that integrating suitable learning strategies can further enhance feature extraction capabilities [46]. Channel attention assigns importance to different channels through weighting, effectively guiding the model’s focus. Building on this foundation, Anwar et al. [33] introduced multiscale Laplacian pyramid attention, Dai et al. [40] introduced second-order channel attention, and Liu et al. [47] introduced spatial attention mechanisms.

Hu et al. [35] pioneered the Squeeze-and-Excitation Network (SENet), which introduced an efficient channel attention mechanism by compressing 2D spatial features into channel weights to explicitly capture interdependencies. While this design significantly improved performance with minimal computational overhead, it relied on aggressive dimensionality reduction that risks discarding fine-grained and clinically important structural features. Building on this foundation, Zhang et al. [36] proposed the Residual Channel Attention Network (RCAN), highlighting the efficacy of channel attention mechanisms in improving feature representation. RCAN was the first to incorporate channel attention mechanisms into residual SR networks, enabling adaptive processing of low- and high-frequency information through the learning of channel-wise interdependence in feature maps. However, its reliance on dimensionality reduction similarly limited the retention of subtle anatomical details. To alleviate this, Wang et al. [48] introduced ECA-Net (Efficient Channel Attention), which removed explicit dimensionality reduction and used a lightweight 1D convolution to capture local cross-channel dependencies while preserving channel information and reducing computational cost. However, the localized design of ECA-Net restricts its ability to capture complex and long-range dependencies that are critical to preserve delicate structural patterns such as early pathological changes.

More recently, Zhang et al. [49] proposed the Squeeze-and-Excitation Reasoning Attention Network (SERAN) that combines channel recalibration with contextual reasoning to enhance the recovery of structural details and improve the accuracy of MRI images. Although SERAN advanced both accuracy and visual quality, it remained limited in addressing high-frequency information loss and complex anatomical structures.

SwinIR [50] Transformer-based model, has shown promise in MRI image SR, due to its ability to handle complex anatomical structures and preserve diagnostic features. However, despite its strength in capturing long-range contextual dependencies, the model can struggle to accurately reconstruct fine local details and high-frequency information, which are essential for preserving minute structural boundaries. Restormer, proposed by Zamir et al. [51], is a Transformer-based architecture employing multi-Dconv head transposed attention (MDTA) and gated-Dconv feed-forward networks (GDFN) to capture long-range dependencies. However, its high computational and memory costs, as well as its limited fine-grained structural recovery in highly textured anatomical regions, pose persistent challenges for MRI SR.

MHAN [52] employs a multi-stage spatial–channel attention cascade to suppress aliasing artifacts and recover high-frequency structural details. However, its limited ability to model diverse features and context significantly reduces its overall performance. In 2024, Hua et al. [53] introduced TDAFD, which incorporates Multi-Scale Feature Distillation (MSFD) blocks coupled with dual-attention mechanisms to enable efficient multi-layer feature extraction. However, its low performance stems from the compounded effect of insufficient feature retention mechanisms, which limit the preservation and recovery of fine contextual information throughout the network’s forward pass.

Hongbi et al. [27] introduced MFER, a model designed to restore degraded high-resolution details through multi-level feature extraction and reconstruction modules, mapping features from each level to the high-resolution space via deconvolution layers. However, its multi-level feature extraction scheme can lead to feature redundancy and ineffective cross-scale feature fusion, and lacks adequate emphasis on informative features during the initial feature extraction stage. Further optimization of the feature extraction module could improve its ability to preserve fine structural details, improve edge sharpness, and minimize artifacts in the reconstructed images.

In 2025, He et al. [54] proposed a dual-channel enhancement model for MRI image SR, using complementary information from different image characteristics. One channel focuses on low-frequency information, capturing overall structure and context, while the other focuses on high-frequency details to enhance edges and textures. However, this separation can lead to information bottlenecks where either global structural coherence is sacrificed for local detail preservation, or fine-grained textures are lost in favor of broader contextual understanding.

The integration of CNNs with specialized attention mechanisms has become a dominant paradigm in biomedical engineering beyond image reconstruction. Prior studies [55,56] showed that hybrid attention–based CNNs significantly improve brain tumor classification by effectively separating pathological features from background noise. In temporal biomedical signal analysis, multi-feature fusion CNNs have been used for epileptic seizure prediction [57,58], highlighting the importance of multi-receptive-field structures. Hybrid architectures have also demonstrated strong robustness in physiological signal tasks, such as ECG authentication (99.7% accuracy) [59] and EEG-based mental-state classification [60].

**Algorithm 1 HACR-Net Architecture**

1: **function** HACR-Net *x* ▷ Input $I_{\text{LR}}\in {\mathbb{R}}^{B\times C_{in}\times H\times W}$; nonlinearity ϕ=PReLU; σ=sigmoid

2:   $f_{0}\leftarrow \phi ({Conv}_{3\times 3}(x))$

3:   $ca\_mask\leftarrow \sigma \;\left({Conv}_{1\times 1}\;\left(\phi \;\left({Conv}_{1\times 1}\;\left(GAP(f_{0})\right)\right)\right)\right)$

4:   $f_{ch}\leftarrow f_{0}⊙ca\_mask$

5:   $sa\_mask\leftarrow \sigma \;\left({Conv}_{7\times 7}\;\left(\phi \;\left({Con9v}_{1\times 1}(f_{ch})\right)\right)\right)$

6:   $f\leftarrow f_{ch}⊙sa\_mask$; $f_{skip}\leftarrow f$

7:  **for** *i* = 1–10 **do**

8:    $f_{input}\leftarrow f$          ▷ Store CAAM input for residual

9:    $res\leftarrow f+{Conv}_{3\times 3}\;\left(\phi \;\left({Conv}_{3\times 3}\;\left(\phi (f)\right)\right)\right)$

10:    $c_{in}\leftarrow {Conv}_{3\times 3}(res)$        ▷ MFAB Block

11:    $f_{a1}\leftarrow {Conv}_{3\times 3}\;\left(\phi \;\left({Conv}_{3\times 3}\;\left(\phi (c_{in})\right)\right)\right)$

12:    $f_{a2}\leftarrow {Conv}_{1\times 1}\;\left(\phi \;\left({Conv}_{3\times 3}\;\left(\phi (c_{in})\right)\right)\right)$

13:    $att\leftarrow \sigma \;\left({Conv}_{1\times 1}\;\left(\phi \;\left({Conv}_{1\times 1}\;\left(GAP(f_{a1}+f_{a2})\right)\right)\right)\right)$

14:    $f_{acr}\leftarrow (f_{a1}+f_{a2})⊙att$

15:    $f_{b}\leftarrow {Conv}_{1\times 1}(c_{in})$

16:    $mfab\_out\leftarrow f_{acr}+f_{b}$        ▷ CRAB Block

17:    $h\leftarrow {Conv}_{3\times 3}(c_{in})$

18:    z←GAP(h)

19:    $u_{1}\leftarrow {Conv}_{1\times 1}^{C\to C_{w}}(z)$

20:    $u_{2}\leftarrow {Conv}_{1\times 1}^{C_{w}\to C_{w}}(u_{1})$

21:    $u_{3}\leftarrow \phi (u_{2})$

22:    $v_{1}\leftarrow {Conv}_{1\times 1}^{C_{w}\to C_{w}}(u_{3})$

23:    $v_{2}\leftarrow {Conv}_{1\times 1}^{C_{w}\to C}(v_{1})$

24:    $crab\_mask\leftarrow \sigma (v_{2})$

25:    crab_out←h⊙crab_mask+h      ▷ Feature Fusion and Residual Connection

26:    $f_{combined}\leftarrow Concat(mfab\_out,crab\_out)$    ▷ Concatenate MFAB and CRAB outputs

27:    $f_{proj}\leftarrow {Conv}_{1\times 1}(f_{combined})$

28:    $f\leftarrow f_{proj}+f_{input}$          ▷ Add CAAM input residual

29:   **end for**

30:   $f_{res}\leftarrow {Conv}_{3\times 3}(f)+f_{skip}$

31:   $f_{↑}\leftarrow PixelShuffle\;\left({Conv}_{3\times 3}(f_{res})\right)$

32:  **return** ${Conv}_{3\times 3}(f_{↑})$          ▷ Output $I_{\text{SR}}$ $\in {\mathbb{R}}^{B\times C_{out}\times rH\times rW}$

33: **end function**

## Methodology

This section provides a comprehensive overview of the proposed HACR-Net architecture. It begins with an outline of the overall network structure, followed by descriptions of its core components HAM, MFAB, and CRAB, and the training objectives.

### Overview of HACR-Net

Fig 1 illustrates the overall architecture of our proposed HACR-Net, which consists of three subnetworks: the shallow feature extraction subnetwork, the deep feature extraction subnetwork, and the reconstruction subnetwork.

Let $I_{\text{LR}}\in {\mathbb{R}}^{C\times H\times W}$ denote the input LR MRI image, where *C* is the number of channels, *H* and *W* are the height and width of the image. The shallow features $F_{1}\in {\mathbb{R}}^{C\times H\times W}$ are extracted using HAM, expressed as:


$$F_{1}={ℋ}_{s}(I_{LR})$$ (1)

where ${ℋ}_{s}(\cdot )$ denotes the operation of shallow feature extraction, and *F*_1_ represents the extracted shallow features. After obtaining shallow feature representation, *F*_1_, we further develop CAAM to extract the deep features denoted as $F_{d}\in {\mathbb{R}}^{H\times W\times C}$. This module is designed to capture richer textures, sharper edges, and semantically coherent features. It integrates a residual block, the MFAB, and CRAB, which collaboratively extract and aggregate features from multiple layers and receptive fields to enhance the overall feature representation, as expressed in the following equation:


$$F_{d}=({ℋ}_{conv}((F_{2},...,F_{k}))+F_{1})$$ (2)

where *F*_*d*_ refers to the high-level MRI feature descriptors extracted by the deep feature subnetwork. The features learned by CAAMs are denoted by $\left(F_{2},...,F_{k}\right)$, where *k* is the total number of CAAM modules. The symbol ’ + ’ denotes the summation operation employed to fuse low-level shallow features with high-level features, thereby contributing to more stable and effective training. In the reconstruction subnetwork, the low-level spatial features *F*_1_, are combined with the high-level semantic features *F*_*d*_, to produce the final super-resolved MRI image. A convolutional layer first processes these fused features, and the resulting feature maps are then upsampled using a PixelShuffle operation, formulated as:


$$I_{\text{SR}}=w_{1}\left(H_{\text{UP}}\left(w_{2}\left(F_{1}+F_{d}\right)\right)\right)$$ (3)

where $I_{\text{SR}}\in {\mathbb{R}}^{C\times H\times W}$ is the reconstructed SR MRI image, H^UP^(.) denotes PixelShuffle layer operation.

### Hybrid attention module (HAM)

To obtain comprehensive and hierarchical shallow features, we designed HAM using channel and spatial attention mechanisms. The channel attention enhances feature maps by adaptively assigning weights to each channel based on importance, while spatial attention directs the network’s focus to spatially significant regions. This design is motivated by the nature of brain MRI images, where anatomical structures and tissue boundaries often span larger areas than in natural images. The larger kernel (7×7) used in the spatial attention provides a wider receptive field, enabling the model to capture broader spatial relationships. By positioning HAM at the shallow feature extraction stage, the module preconditions the input representation, ensuring that salient structural boundaries, tissue interfaces, and subtle pathological regions are emphasized before deeper processing. This early refinement reduces the propagation of irrelevant background features, thereby enhancing the efficiency and accuracy of subsequent CAAMs.

Let $I_{\text{LR}}\in {\mathbb{R}}^{C\times H\times W}$ denote the LR input image, processed through a 3×3 convolution layer to extract feature *F*, which is subsequently fed into a channel attention block to obtain channel attention map, *F*_*c*_ is computed as:


$$F_{c}=\sigma \left({Conv}_{1\times 1}^{\left(2\right)}\left(\delta \left({Conv}_{1\times 1}^{\left(1\right)}\left(GAP(F)\right)\right)\right)\right)$$ (4)

$$GAP=\frac{1}{W\times H}\sum_{i=0}^{H}\sum_{j=0}^{W}F(i,j)$$ (5)

$$F^{\prime }=F_{c}⊗F$$ (6)

where, GAP(·) denotes global average pooling, ${Conv}_{1\times 1}^{\left(1\right)}$ and ${Conv}_{1\times 1}^{\left(2\right)}$ represent the first and second 1×1 convolutional layers respectively, δ(·) is the PReLU activation function, and σ(·) is the sigmoid activation function used to normalize the attention weights. The GAP layer extracts channel-wise descriptors by averaging spatial information, capturing the general characteristics of each channel. ${Conv}_{1\times 1}^{\left(1\right)}$ layer then reduces the channels to *C*/*r*, followed by PReLU for non-linearity. ${Conv}_{1\times 1}^{\left(2\right)}$ restores the channels to *C*, and a sigmoid activation normalizes the attention weights to obtain *F*_*c*_. The refined feature map is obtained by multiplying *F*_*c*_ and *F* as shown in equation (6). These help the model focus on the most informative feature channels. The spatial attention block models spatial dependencies in feature maps, highlighting informative regions to improve fine detail. The spatial attention map, *M*_*s*_, is given by Equation (7) as:


$$M_{s}=\sigma \left({Conv}_{1\times 1}\left(\delta \left({Conv}_{7\times 7}\left({Conv}_{1\times 1}(F^{\prime })\right)\right)\right)\right)$$ (7)

$$F_{1}=M_{s}⊗F^{\prime }$$ (8)

The refined feature map *F*_1_ is obtained by multiplying *M*_*s*_ by *F*′, integrating spatial and channel-wise attention. This enables a comprehensive and hierarchical extraction of shallow features.

### Context-aware aggregation module (CAAM)

CAAM integrates MFAB and CRAB with a residual block for deep feature reuse and efficient gradient flow. Together, these components capture and aggregate features at multiple scales while preserving essential structural and semantic information. MFAB processes inputs through parallel convolutional branches with varying kernel sizes to capture features across different receptive fields, combining fine-grained local details with broader structural patterns. CRAB complements this process by employing channel retention and attention strategies that maintain broader channel width, thereby enhancing feature channels and preserving critical information. The outputs of MFAB and CRAB are adaptively fused, ensuring effective integration of low- and high-level information.

Let *F*_1_ denote the CAAM input, with *N* representing the number of CAAM modules; the output of the N−th CAAM can be expressed as:


$$F_{\text{MFAB}}=f_{\text{MFAB}}\left(f_{\text{Conv}}\left(F_{N-1}\right)\right)$$ (9)

$$F_{\text{CRAB}}=f_{\text{CRAB}}\left(f_{\text{Conv}}\left(F_{N-1}\right)\right)$$ (10)

$$F_{\text{CAAM}}=f_{\text{Concat}}\left(F_{\text{MFAB}},F_{\text{CRAB}}\right)+F_{N-1}$$ (11)

where $f_{\text{MFAB}}(\cdot )$ denotes the function of feature extraction through the MFAB, $f_{\text{CRAB}}(\cdot )$ denotes the function of feature extraction through the CRAB, and $f_{\text{Concat}}(\cdot ,\cdot )$ denotes feature fusion. *F*_*N*_ represents the feature obtained by the operation of the Nth CAAM.

### Multiscale feature aggregation block (MFAB)

**Fig 2:**
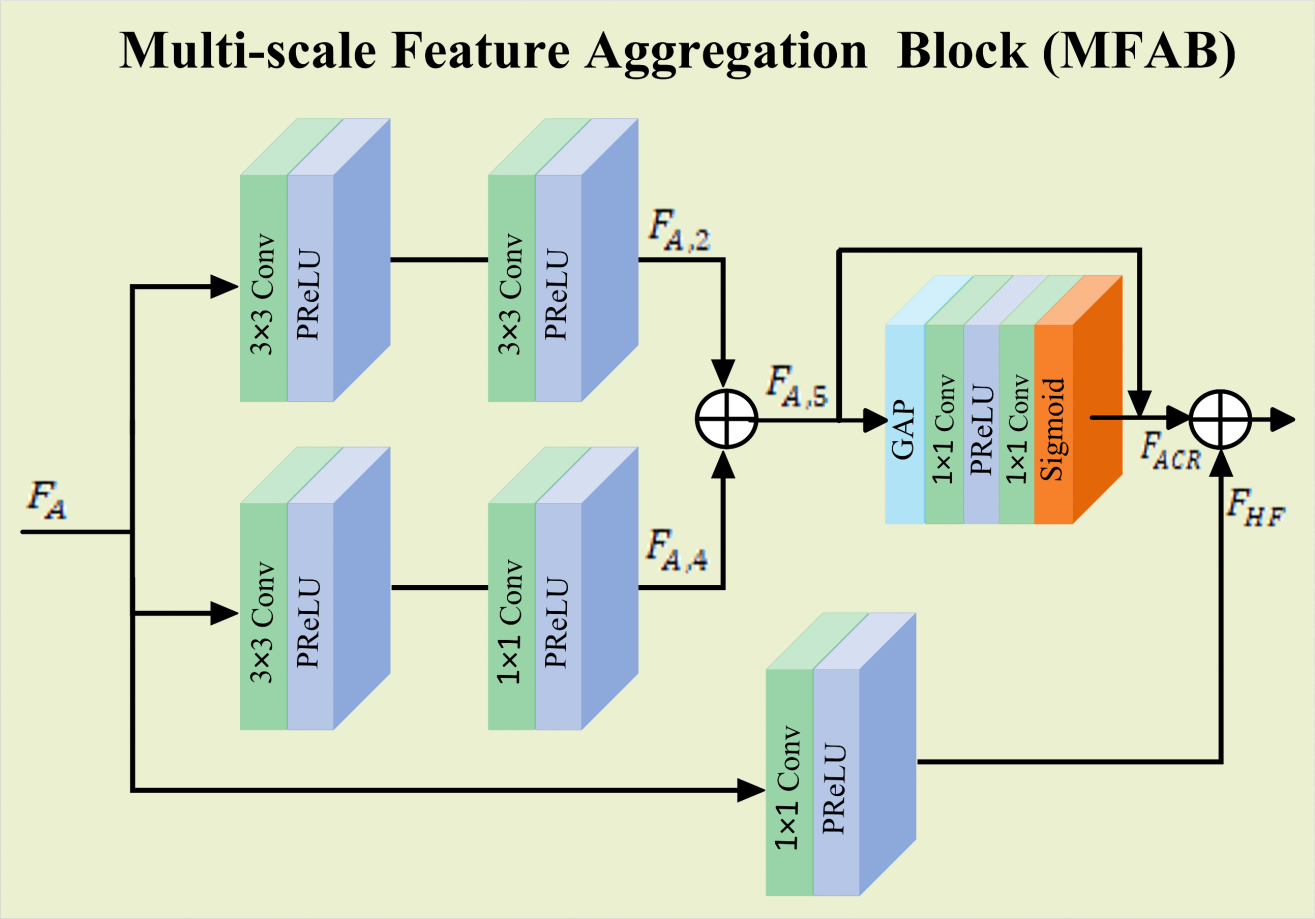
Multiscale Feature Aggregation Block (MFAB).

The Multiscale Feature Aggregation Block (MFAB), illustrated in Fig 2 is designed to extract and aggregate features using different convolution layers with varying receptive fields. This design enables the network to capture and integrate essential features across multiple spatial scales. To achieve this, MFAB employs convolution with kernels of different sizes to extract multiscale features, which are subsequently combined by element-wise summation. An adaptive channel refinement step then dynamically re-weights the aggregated features according to their relative importance. This refinement is particularly advantageous for MRI data, where tissue structures such as white matter and gray matter exhibit varying levels of textural complexity. In contrast to conventional methods such as MSRN [21] and RDN [20], which rely on simple concatenation or summation, the proposed adaptive refinement prioritizes the most salient features for reconstructing fine anatomical details, making the aggregation process more effective and context-aware.

Initially, two sequential convolution layers with 3×3 kernels and PReLU activation are applied to capture the fundamental spatial features of the input feature map.


$$F_{A,2}=\delta \left({\mathrm{Conv}}_{3\times 3}\left(\delta \left({\mathrm{Conv}}_{3\times 3}(F_{A})\right)\right)\right)$$ (12)

Afterward, the process continues with two additional convolutional layers: one with a 3×3 kernel and another with a 1×1 kernel, both of which have PReLU activation, further facilitating the extraction of multiscale information.


$$F_{A,4}=\delta \left({\mathrm{Conv}}_{1\times 1}\left(\delta \left({\mathrm{Conv}}_{3\times 3}(F_{A})\right)\right)\right)$$ (13)

The outputs from these pathways are fused through element-wise summation, ensuring effective feature integration across different receptive fields. To further refine feature representations, we incorporate an adaptive channel refinement to enable the network to emphasize the most relevant information. Furthermore, a 1×1 point-wise convolution layer with PReLU activation is employed to capture fine-grained details and enhance high-frequency information.


$$F_{MFAB}=F_{ACR}+F_{HF}$$ (14)

By integrating convolution layers with varying receptive fields and an adaptive channel refinement, MFAB enables effective multiscale feature learning.

### Channel retention attention block (CRAB)

**Fig 3:**
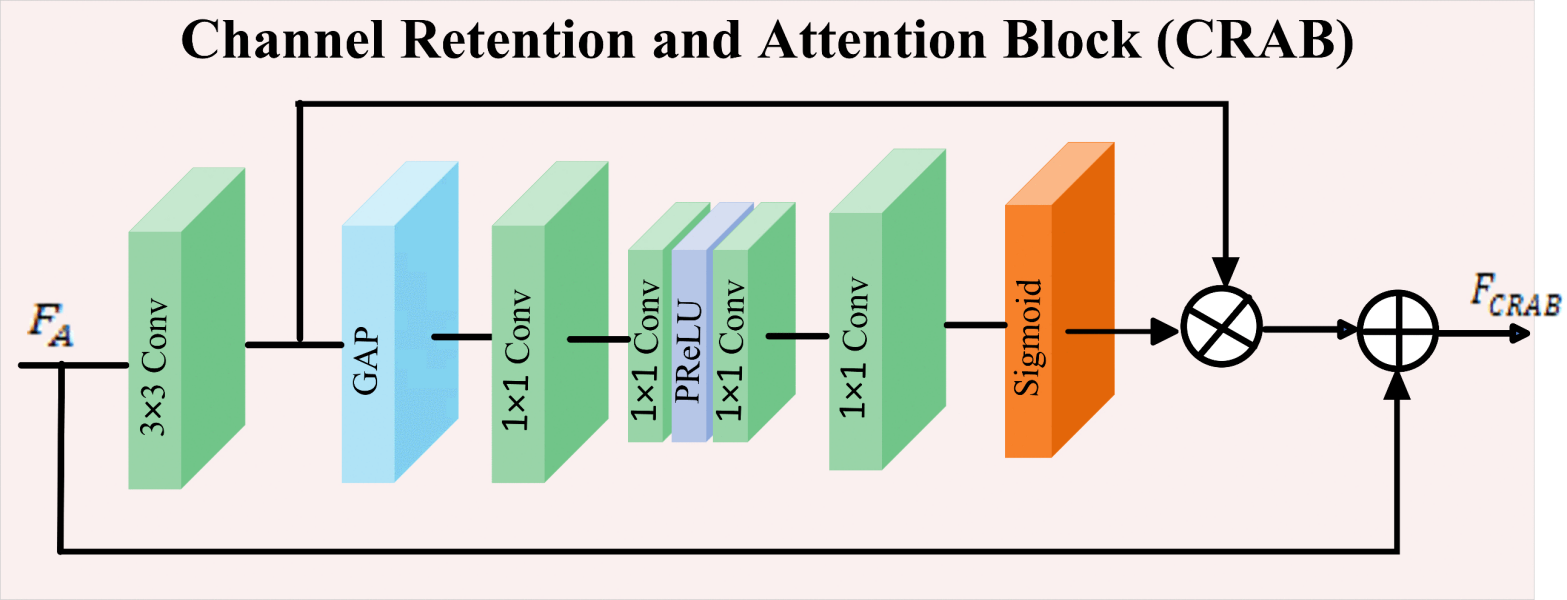
Channel Retention and Attention Block (CRAB).

As illustrated in Fig 3, the Channel-Retention Attention Block (CRAB) is designed to mitigate the loss of fine-grained anatomical information that often occurs during channel compression in deep networks. Conventional attention modules exacerbate this issue by applying aggressive compression that discards subtle features. To address this, CRAB employs a channel retention strategy that preserves contextual details through the intermediate channel width.

Let the input feature map be denoted as $F_{A}\in {\mathbb{R}}^{C\times H\times W}$, where *C*, *H*, and *W* represent the number of channels, height, and width, respectively. CRAB begins by applying a 3×3 convolution to extract local spatial features. These features are then globally aggregated using GAP to form a compact channel descriptor that captures the global context of each feature channel. To model inter-channel dependencies while preserving representational richness, the descriptor is passed through a reduction–expansion bottleneck structure. Unlike classical SE blocks [35,61], CRAB avoids overly narrow intermediate projections. The ${\text{Conv}}_{1\times 1}^{b}$ and ${\text{Conv}}_{1\times 1}^{c}$ layers maintain a relatively wide channel width, reducing the risk of suppressing subtle but informative anatomical features. Attention weights are normalized using a sigmoid function, enabling independent reweighting of each channel, computed in Eq. (15) as:


$$F_{CRAB}=({Conv}_{3\times 3}(F_{A})\cdot \sigma ({Conv}_{1\times 1}^{d}({Conv}_{1\times 1}^{c}(\delta ({Conv}_{1\times 1}^{b}({Conv}_{1\times 1}^{a}(GAP(F_{A})))))))))+F_{A}$$ (15)

where, ${\text{Conv}}_{3\times 3}$ extracts spatial features, ${\text{Conv}}_{1\times 1}^{a}$ reduces dimensionality, ${\text{Conv}}_{1\times 1}^{b}$ and ${\text{Conv}}_{1\times 1}^{c}$ act as retained-width bottlenecks, and ${\text{Conv}}_{1\times 1}^{d}$ restores the original channel dimension. δ denotes the PReLU activation, and σ represents sigmoid activation. The residual addition with *F*_*A*_ ensures that original semantic information is preserved while the attention mechanism refines salient features.

### Training objectives

HACR-Net aims to establish an end-to-end mapping $F_{\text{HACR-Net}}$, between the LR image and the HR image. This is achieved by optimizing the model’s parameters to minimize the loss between the reconstructed image and the ground truth. Using a training dataset $\{I_{i}^{\text{LR}},I_{i}^{\text{HR}}{\}}_{i}^{N}$, where *N* represents the total number of images in the training set, the most commonly used loss functions in SR are the mean square error (MSE), *L*1 and the mean absolute error (MAE), *L*2. Although these approaches can enhance PSNR, they often result in overly smoothed high-frequency details, adversely affecting the visual quality of the reconstructed image. Advanced loss functions have been introduced in natural image SR to enhance performance. However, when applied to medical images, methods such as perceptual loss [62], Charbonnier loss [63], and adversarial loss [64] can cause distortions in texture and structure, potentially compromising diagnostic accuracy and further analysis. To ensure a fair comparison with prior methods, the *L*1 loss function was adopted to guide model optimization.


$$L(\theta )=\frac{1}{\left|N\right|}\sum_{i=1}^{\left|N\right|}\|I_{i}^{\text{HR}}-F_{\text{HACR-Net}}(I_{i}^{\text{LR}};\theta ){\|}_{1}$$ (16)

where θ represents the learnable parameters of our network, $I_{i}^{\text{HR}}$ is the ground truth corresponding to $I_{i}^{\text{LR}}$, and $F_{\text{HACR-Net}}(\cdot )$ refers to the overall function of our proposed HACR-Net network. In the experiment, training with degraded MRI samples and using *L*1 results in faster and more stable convergence during the training process.

### Experiment

This section presents a comprehensive evaluation of the proposed method. The datasets used in the experiments were first introduced, followed by a detailed description of the implementation procedures and the evaluation metrics employed. The performance of our proposed method is compared with several state-of-the-art approaches to highlight its effectiveness and practical relevance. Finally, an ablation study is presented that investigates the contribution of each component in the network.

### Dataset

To ensure the robustness and generalizability of the proposed approach, two benchmark datasets were utilized. Each dataset is partitioned into 70% for training, 10% for validation, and 20% for testing.

**IXI Dataset:** Available at IXI Dataset, comprises 578 PD volumes, 581 T1 volumes, and 578 T2 volumes. Each volume has a dimension of 256×256×96 (height, width, depth), where 96 is the number of slices in each MRI volume.
**BraTS2018:** The Brain Tumor Segmentation 2018 Challenge dataset [65,66] consists of 285 cases, 210 cases of high-grade glioma (HGG) and 75 cases of low-grade glioma (LGG). Each MRI sequence has dimensions of 240 × 240 pixels with a total of 155 slices per volume.

The IXI dataset includes scans acquired across three hospitals using scanners from different manufacturers at both 1.5T and 3T field strengths, capturing variability in acquisition conditions. BraTS2018 comprises multi-institutional data with heterogeneous scanner protocols, and importantly, includes pathological cases (brain tumors), reflecting real-world clinical scenarios. Together, these datasets cover healthy and diseased tissue, multiple field strengths, diverse acquisition protocols, and multi-institutional variability, supporting the representativeness of our evaluation for real-world MRI scans.

The LR images are obtained by applying 3×3 Gaussian filter with a standard deviation of 1 to the HR images, followed by bicubic downsampling with scale factors of ×2 and ×4. This degradation procedure aligns with the approach adopted in [67,68], which employed a similar strategy to generate LR MRI brain images in the spatial domain. Before training all intensity values in the datasets are normalized to a range of [0–1]. To ensure rigorous and fair comparison, we adhered to a standardized evaluation protocol. Fixed train/validation/test splits were consistently applied across all methods. The quantitative and qualitative results presented were obtained using the same independent test set and identical evaluation scripts as those used from their published models. This uniform setup ensures that performance differences reflect the intrinsic capabilities of each method under similar testing conditions. The subject and volume IDs for the splits are provided in our repository.

### Implementation details and evaluation metrics

The proposed HACR-Net was implemented in the PyTorch 2.0 framework and trained on an NVIDIA RTX 3090 Ti GPU. The Adam optimizer [69] was employed with parameters $\beta_{1}=0.9$, $\beta_{2}=0.999$, and $\epsilon =10^{-8}$, using an initial learning rate of $1\times 10^{-4}$. The HACR-Net architecture comprises 10 CAAMs, each containing one CRAB and one MFAB. Training was conducted for 250 epochs with a batch size of 32.

The model was quantitatively evaluated using two widely adopted image quality metrics: peak signal-to-noise ratio (PSNR) and structural similarity index measure (SSIM). PSNR measures the ratio, on a logarithmic scale, between the maximum possible pixel value and the mean squared error (MSE) of the reconstructed image with respect to the ground-truth, as expressed in equation (17). Higher PSNR values indicate lower reconstruction error and greater fidelity to the reference image. SSIM complements PSNR by evaluating perceptual image quality through the joint assessment of luminance, contrast, and structural information [70]. Unlike pixel-wise error metrics, SSIM captures spatial dependencies between pixels, yielding a measure that is more consistent with human visual perception. Both metrics were computed across all test samples, and the results are reported as mean values to ensure a fair comparison.

PSNR and SSIM often reflect only statistical similarity between the reconstructed image and the ground truth. To address this limitation, we additionally employed the LPIPS metric, which provides a perceptual similarity assessment aligned with human visual judgment [71]. LPIPS computes deep feature embeddings from networks pre‑trained on large‑scale image datasets, capturing high‑level perceptual attributes that better reflect the visibility of anatomical structures and clinically relevant details [72]. This makes the combined metrics more representative of real‑world diagnostic relevance.


$$\text{PSNR}=10\log_{10}\left(\frac{(2^{n}-1{)}^{2}}{\text{MSE}}\right)$$ (17)

where *n* represents the image’s bit depth (e.g., 8 for an 8-bit image), $(2^{n}-1{)}^{2}$ corresponds to the square of the maximum possible pixel value. MSE (Mean Squared Error) measures the reconstruction error, with smaller values indicating a closer match between the reconstructed image and the ground truth. Higher PSNR values indicate better image reconstruction quality.


$$\text{SSIM}=\frac{\left(2\mu_{x}\mu_{y}+C_{1})(2\sigma_{x}\sigma_{y}+C_{2}\right)}{\left(\sigma_{x}^{2}+\sigma_{y}^{2}+C_{2})(\sigma_{x}^{2}+\sigma_{y}^{2}+C_{2}\right)}$$ (18)

where $\mu_{x}$ and $\mu_{y}$ are the local means, $\sigma_{x}$ and $\sigma_{y}$ are the standard deviations, $\sigma_{xy}$ is the cross-covariance for images *x* and *y*, respectively, and *C*_1_ and *C*_2_ are constant terms.


$$\text{LPIPS}(x,y)=\sum_{l}\frac{1}{H_{l}W_{l}}\sum h,w{\left\|w_{l}⊙\left({\hat{f}}_{l}(x{)}_{hw}-{\hat{f}}_{l}(y{)}_{hw}\right)\right\|}_{2}^{2}$$ (19)

where *H*_*l*_ and *W*_*l*_ denote the height and width of the input feature at layer *l* in the pretrained network. The term *w*_*l*_ corresponds to the learned weight assigned to each channel in layer *l*. ${\hat{f}}_{l}(x{)}_{hw}$ and ${\hat{f}}_{l}(y{)}_{hw}$ represent the normalized feature vectors at spatial locations (*h*, *w*) for images *x* and *y*, respectively. The operator ⊙ indicates element-wise multiplication between the feature vectors and their associated weights.

## Results and discussion

This subsection presents a comprehensive evaluation of the proposed method through quantitative and qualitative analyses. The quantitative assessment uses PSNR, SSIM, and LPIPS to compare the performance of the proposed method with existing approaches. The qualitative analysis involves visual inspection, focusing on perceptual quality and structural fidelity.

### Quantitative comparison

In this subsection, we provide a comprehensive quantitative evaluation of the proposed HACR-Net and compare its performance with that of state-of-the-art SR methods. The compared methods include SRCNN [18], VDSR [19], EDSR [73], RDN [20], RCAN [36], CSN [14], SERAN [49], SwinIR [50], Restormer [51], MFER [27], and SenseSR [54]. Table 1 presents the quantitative results of the compared methods on the BraTS 2018 dataset, while Table 2 presents the results on the IXI dataset. The proposed HACR-Net consistently outperforms state-of-the-art methods, achieving superior SR performance across the two datasets at scaling factors of ×2 and ×4.

**Table 1:** Average PSNR, SSIM and LPIPS comparison on the BraTS 2018 dataset at ×2 and ×4 scaling factor.

	×2			×4		
Methods	PSNR↑	SSIM↑	LPIPS↓	PSNR↑	SSIM↑	LPIPS↓
Bicubic	30.92	0.8601	0.3151	25.87	0.8388	0.4419
SRCNN [18]	31.66	0.9065	0.2411	28.18	0.8835	0.2552
VDSR [19]	34.86	0.9840	0.1767	28.74	0.9423	0.2587
EDSR [73]	33.26	0.9466	0.2025	30.25	0.9023	0.2569
RDN [20]	34.57	0.9536	0.1580	30.92	0.9172	0.2558
RCAN [36]	34.82	0.9556	0.1556	33.11	0.9301	0.1605
CSN [14]	36.33	0.9582	0.1338	33.21	0.9403	0.1563
SERAN [49]	38.77	0.9625	0.1160	33.22	0.9419	0.1552
SwinIR [50]	38.15	0.9785	0.1022	33.34	0.9427	0.1555
Restormer [51]	38.92	0.9892	0.1021	33.40	0.9451	0.1547
MFER [27]	40.45	0.9890	0.0958	33.69	0.9465	0.1534
SenseSR [54]	*42.78*	*0.9894*	*0.0903*	*33.91*	*0.9523*	*0.1501*
**HACR-Net (Ours)**	**43.51**	**0.9895**	**0.0822**	**34.80**	**0.9552**	**0.1475**

**Table 2:** Performance comparison of average PSNR, SSIM, and LPIPS on different SR methods across IXI (PD, T1, and T2) dataset.

	PD						T1						T2					
	×2			×4			×2			×4			×2			×4		
Method	PSNR↑	SSIM↑	LPIPS↓	PSNR↑	SSIM↑	LPIPS↓	PSNR↑	SSIM↑	LPIPS↓	PSNR↑	SSIM↑	LPIPS↓	PSNR↑	SSIM↑	LPIPS↓	PSNR↑	SSIM↑	LPIPS↓
Bicubic	35.04	0.9664	0.2847	29.13	0.8799	0.4563	33.80	0.9525	0.3037	28.28	0.8312	0.4637	33.44	0.9589	0.3016	27.86	0.8611	0.4619
SRCNN [18]	38.96	0.9861	0.1222	31.11	0.9181	0.2567	37.12	0.9761	0.1318	29.90	0.8796	0.2638	37.32	0.9796	0.1364	29.69	0.9052	0.2664
VDSR [19]	39.97	0.9861	0.1048	32.09	0.9311	0.2218	37.67	0.9783	0.1249	30.57	0.8932	0.2577	38.65	0.9836	0.1305	30.79	0.9240	0.2581
EDSR [73]	39.87	0.9857	0.1051	31.80	0.9284	0.2393	37.56	0.9774	0.1255	30.46	0.8902	0.2593	38.28	0.9824	0.1198	30.52	0.9198	0.2575
RDN [20]	40.31	0.9870	0.1019	32.73	0.9387	0.2130	37.95	0.9795	0.1236	31.05	0.9042	0.2552	38.75	0.9838	0.1171	31.45	0.9324	0.2388
RCAN [36]	40.57	0.9871	0.1011	32.58	0.9367	0.2136	37.88	0.9793	0.1238	30.85	0.8965	0.2590	39.24	0.9841	0.1119	31.60	0.9298	0.2360
CSN [14]	41.28	0.9895	0.0990	33.40	0.9486	0.2052	38.27	0.9810	0.1192	31.23	0.9093	0.2419	39.71	0.9863	0.1052	32.05	0.9413	0.2213
SERAN [49]	41.53	0.9900	0.0982	33.72	0.9526	0.2019	38.66	0.9822	0.1185	31.89	0.9201	0.2382	40.18	0.9872	0.1012	32.40	0.9455	0.2140
SwinIR [50]	41.48	0.9889	0.0976	33.63	0.9532	0.2017	38.67	0.9825	0.1184	32.08	0.9224	0.2350	40.11	0.9868	0.1003	32.44	0.9478	0.2128
Restormer [51]	41.72	0.9893	0.0954	33.70	0.9540	0.2022	38.71	0.9829	0.1160	32.12	0.9235	0.2239	40.09	0.9881	0.0999	32.53	0.9481	0.2137
MFER [27]	41.98	0.9901	0.0924	33.79	0.9541	0.2015	38.78	0.9833	0.1161	32.22	0.9271	0.2218	40.25	0.9873	0.1016	32.72	0.9488	0.2140
SenseSR [54]	*42.00*	*0.9903*	*0.0883*	*33.80*	*0.9548*	*0.1854*	*38.93*	*0.9840*	*0.1103*	*32.34*	*0.9277*	*0.2117*	*40.63*	*0.9875*	*0.0996*	*32.75*	*0.9496*	*0.2119*
**HACR-Net (Ours)**	**42.10**	**0.9907**	**0.0835**	**33.88**	**0.9583**	**0.1786**	**39.34**	**0.9867**	**0.1044**	**32.71**	**0.9308**	**0.2014**	**41.15**	**0.9891**	**0.0981**	**32.89**	**0.9573**	**0.2001**

**Fig 4:**
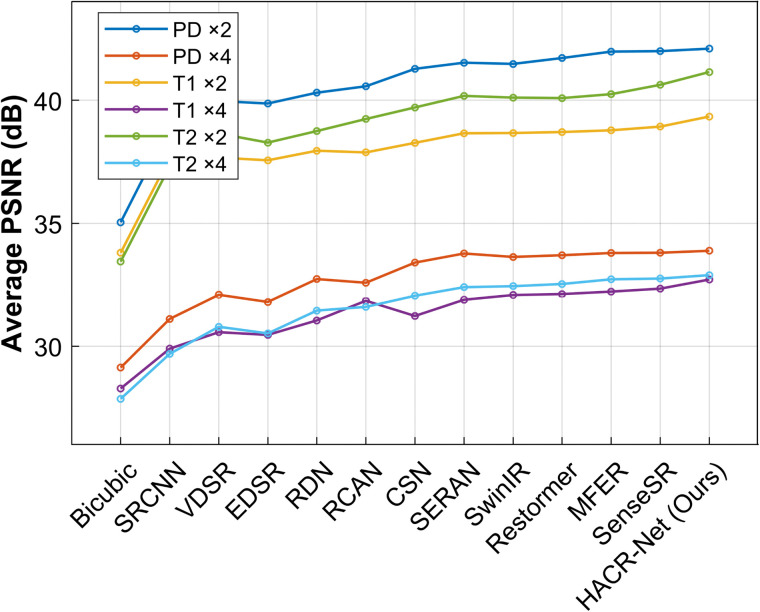
Average PSNR comparison for PD, T1, and T2 modalities of IXI dataset test based on ×2 and ×4 enlargement factors.

**Fig 5:**
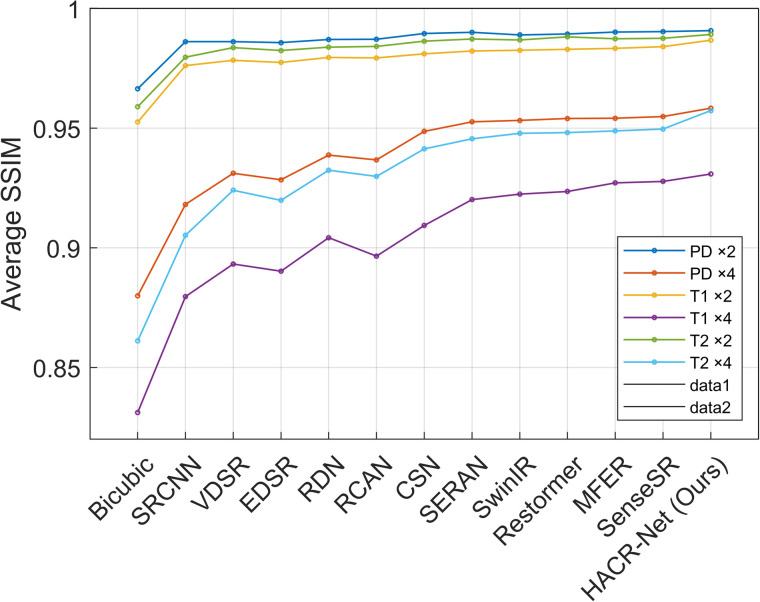
Average SSIM comparison for PD, T1, and T2 modalities of IXI dataset test based on ×2 and ×4 enlargement factors.

**Fig 6:**
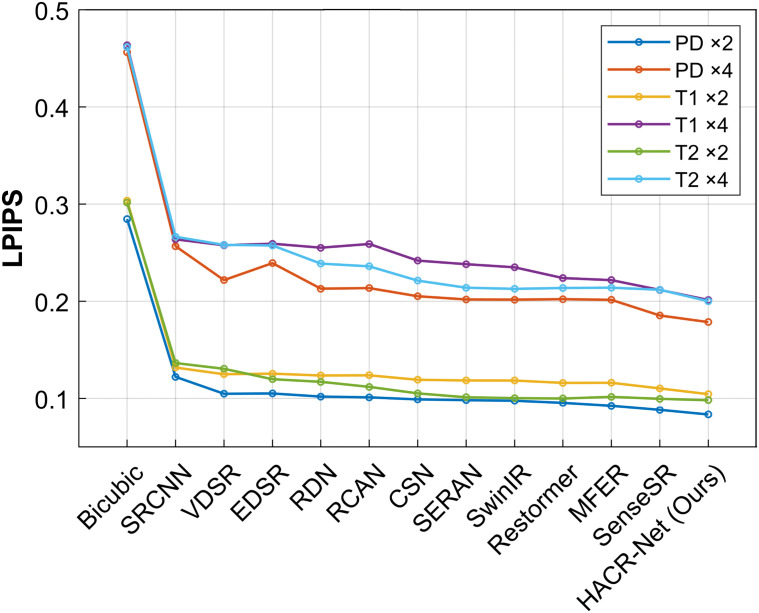
Comparison of LPIPS performance across state-of-the-art models for PD, T1, and T2 modalities of IXI test dataset, evaluated at ×2 and ×4 upscaling factors.

On the BraTS 2018 dataset, HACR-Net demonstrates strong robustness, providing high-fidelity reconstructions, and maintaining competitive accuracy on the ×4 scale. Figs 4–6 present the PSNR, SSIM and LPIPS comparisons between the proposed method and various state-of-the-art approaches on the IXI dataset. At the ×4 scaling factor, HACR-Net achieves PSNR values of 33.88 dB, 32.71 dB, and 32.89 dB for PD, T1, and T2, respectively, surpassing the second-best method, SenseSR, which records 33.80 dB, 32.34 dB, and 32.75 dB on the same modalities. This improvement highlights HACR-Net’s robustness in reconstructing fine anatomical details and complex textures under challenging upscaling conditions. For the BraTS 2018 dataset, HACR-Net achieves a PSNR of 43.51 dB at ×2 upscaling, surpassing the second-best method, SenseSR, which attains 42.78 dB. Similar improvements are evident at ×4 upscaling, where HACR-Net attains 34.80 dB, outperforming the second-best method’s 33.91 dB. The best results are in bold, and the second-best are in italics.

HACR-Net achieves a 0.73 dB improvement in PSNR over the second-best method, SenseSR, on the BraTS 2018 dataset at ×2 (Table 1). This represents a meaningful reduction in reconstruction error, not a marginal gain. The LPIPS score of 0.0822, compared to SenseSR’s 0.0903, further demonstrates that HACR-Net produces reconstructions that are perceptually closer to the ground truth. This is especially important in clinical applications, where diagnostic reliability depends on both pixel-level accuracy and perceptual fidelity.

As shown in Table 2, for a ×2 scaling factor, HACR-Net achieves PSNR/SSIM scores of 42.10 dB / 0.9907 for the PD modality, 39.34 dB / 0.9867 for the T1 modality, and 41.15 dB / 0.9891 for the T2 modality on the IXI dataset with a low LPIPS score. Compared to Restormer [51], MFER [27], and SenseSR [54], HACR-Net shows consistent improvements in PSNR and SSIM in all the magnification factors evaluated. This performance is attributed to the integration of the HAM module in the shallow layers and the CAAM module in the deep feature extraction stage, which enables the preservation of diagnostically relevant information and enhances the reconstruction quality of fine anatomical structures, including cortical boundaries and white matter tracts.

### Qualitative comparison

**Fig 7:**
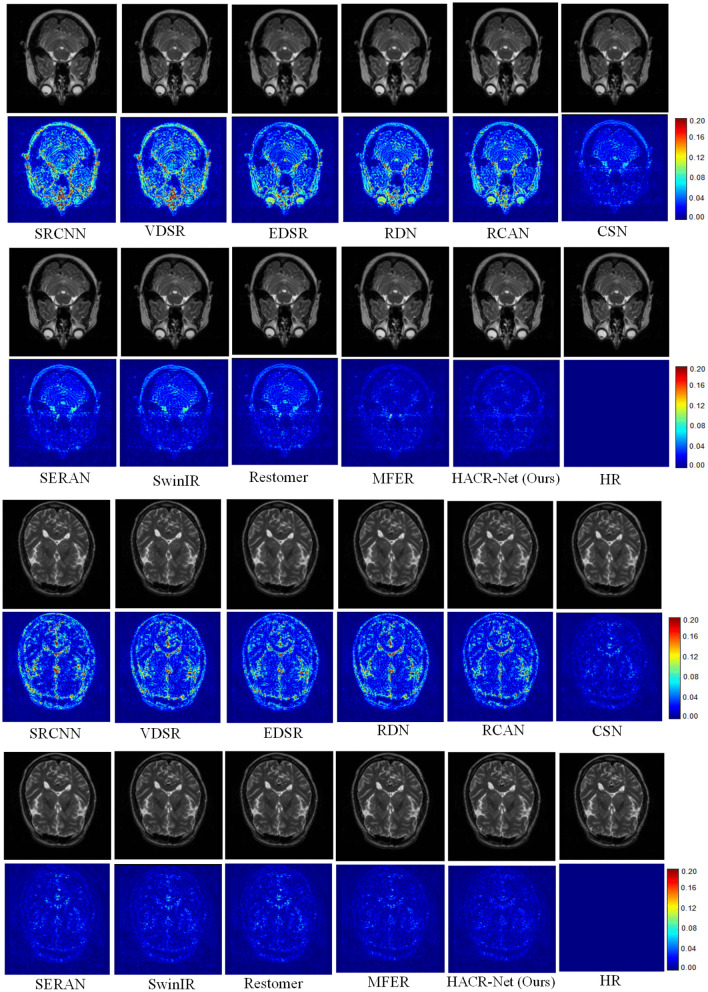
Visual comparison of reconstruction results from different methods on the IXI dataset at × 4 scaling. The corresponding error maps highlight differences in reconstruction accuracy.

The qualitative results presented in Fig. 7 provide direct visual validation of the quantitative results reported in Tables 1 and 2. The error maps generated by the proposed HACR-Net exhibit smoother distributions compared to those of the competing methods. This smoothness is consistent with the higher PSNR and SSIM scores achieved across all test cases. The reconstruction improvements are clearly visible along complex structural edges and low-contrast boundary regions.

Fig 7 shows the visual comparison of our method with other state-of-the-art approaches, along with their corresponding error maps. Regions with more texture indicate higher reconstruction errors, whereas smoother areas reflect improved accuracy. The competing methods concentrate errors around high-frequency structural edges and low-contrast boundaries, whereas HACR-Net minimizes reconstruction errors and maintains structural fidelity in these critical regions. This improvement is driven by the roles of MFAB, which aggregates features across multiple receptive fields to capture fine-grained and global structural patterns, and CRAB, which preserves anatomical context while mitigating information loss from aggressive dimensionality compression, unlike SERAN, which visibly shows distortion around fine anatomical regions.

**Fig 8:**
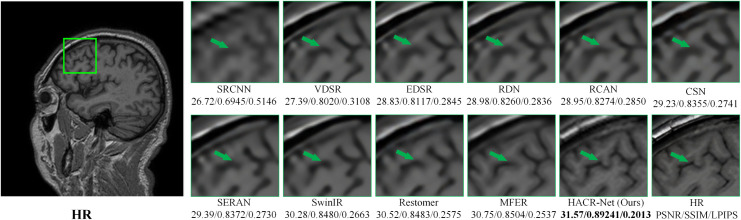
Visual comparison of different SR methods on the T1-weighted IXI dataset at × 4 magnification. The green box indicates the zoomed-in region, where local textures and fine anatomical structures can be clearly assessed.

**Fig 9:**
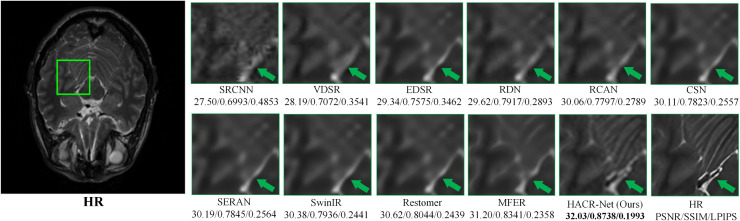
Visual comparison of different SR methods on the T2-weighted IXI dataset at × 4 magnification. The green box highlights the zoomed-in region, enabling evaluation of structural fidelity and reconstruction detail.

**Fig 10:**
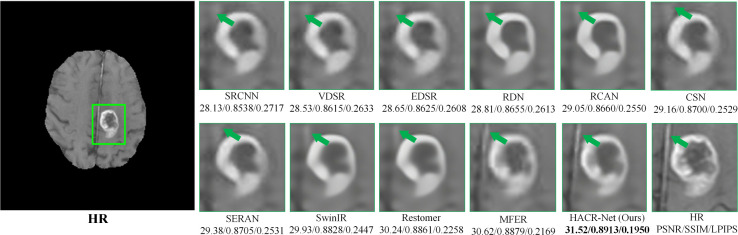
Visualization of reconstruction results from different methods on the BraTS 2018 dataset at × 4 scaling. The green box highlights the zoomed-in region, where edge sharpness and boundary preservation can be clearly observed.

As shown in Fig 8, HACR-Net appears to preserve the fine cortical folding of the frontal lobe (green arrow) more effectively and maintains the connectivity of nearby ridges by addressing suboptimal feature representations at the initial stage using HAM. However, other methods, such as SwinIR and Restormer, exhibit partial discontinuities and smoothing in these regions. Fig 9 shows that HACR-Net maintains a clear separation of adjacent tissue types and preserves structural transitions. At the same time, other approaches, such as Restormer and MFER, tend to merge or blur tissue boundaries. Extending this strength to pathological regions shown in Fig 10, HACR-Net demonstrates improved sharpness at tumor boundaries with sharper delineation while suppressing halo artifacts, as observed in competing methods.

This performance stems from HACR-Net’s enhanced feature aggregation, which enables the recovery of high-frequency edge information while preserving global anatomical coherence. The visual comparisons further confirm that the observed improvements in reconstructed images are both meaningful and directly attributable to the network’s specialized architecture. Specifically, the HAM module effectively preserves shallow structural features (Fig 8, green arrow), while the CRAB module enhances fine contextual details (Fig 9, clear tissue separation). Moreover, these components jointly contribute to greater robustness against input degradation by emphasizing anatomically relevant regions and stabilizing intermediate feature representations. These qualitative improvements are consistent with the quantitative gains summarized in Tables 1 and 2, collectively validating the method’s performance and resilience under adverse imaging conditions.

### Complexity analysis

**Fig 11:**
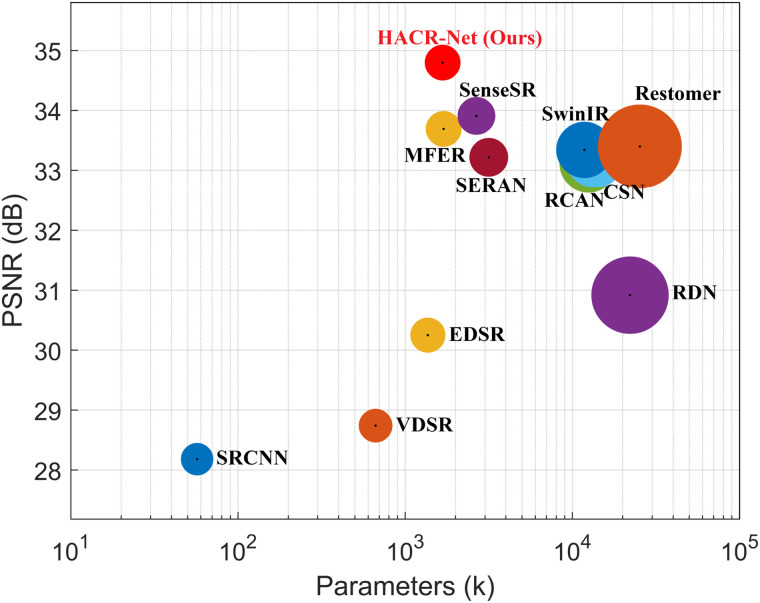
Comparison of PSNR performance versus model parameters on the BraTS2018 dataset at ×4 magnification.

**Fig 12:**
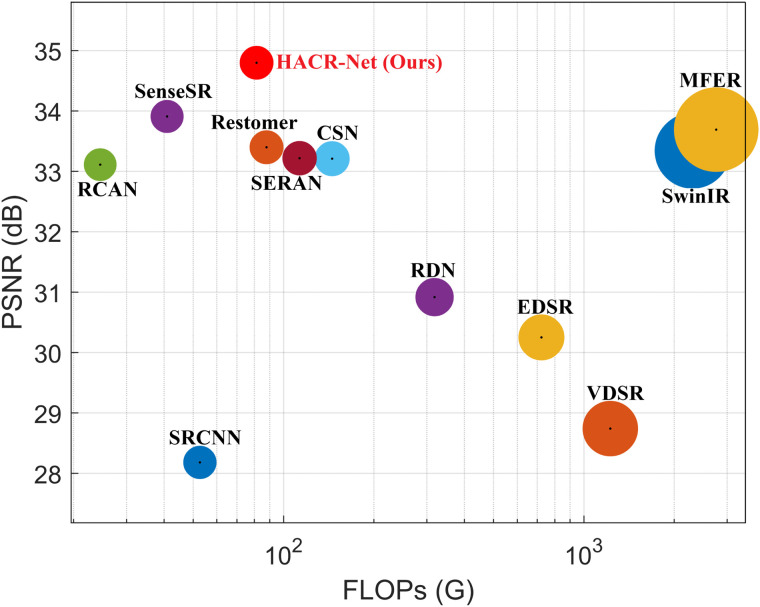
Comparison of PSNR and FLOPs on the BraTS 2018 dataset ×4 magnification.

HACR-Net demonstrates exceptional computational efficiency and scalability. As shown in Table 3, it achieves 1,674k parameters with 81.3G FLOPs and 1.25s inference time on T2-weighted IXI scans in terms of speed and resource usage. Its modular CAAM architecture further enables flexible scaling: reducing the number of CAAMs from 10 to 7 results in minimal PSNR degradation (32.61 vs. 32.89dB) while proportionally reducing parameters, with computational cost scaling linearly with network depth (Table 4). This demonstrates that HACR-Net preserves reconstruction quality under constrained computational budgets and can be flexibly adapted to varying input resolutions. Performance and complexity trade-offs on the BraTS 2018 dataset are further illustrated in Fig 11 and Fig 12.

**Table 3:** Parameters, FLOPs and average running time on T2-weighted IXI dataset at ×2 magnification.

Methods	Parameters (k)	FLOPs	Inference (s)
SRCNN [18]	57	52.7G	1.76
VDSR [19]	665	1225.2G	3.45
EDSR [73]	3,1400	722.61G	2.37
RDN [20]	22,120	318.4G	2.15
RCAN [36]	12,460	24.5G	1.60
CSN [14]	13,640	145.2G	1.85
SERAN [49]	3,160	113.0G	1.17
SwinIR [50]	11,800	2301.0G	1.80
Restormer [51]	25,310	87.7G	1.10
MFER [27]	1,700	2761.0G	1.92
**HACR-Net (Ours)**	**1,674**	**81.3G**	**1.25**

**Table 4:** Ablation study on the effect of varying CAAM module numbers on the IXI T2 dataset at ×4 magnification.

Number of CAAMs	PSNR	SSIM	LPIPS	Parameters (k)
7	32.61	0.9528	0.2103	1670.8
8	32.62	0.9533	0.2080	1672.5
9	32.67	0.9537	0.2035	1673.1
10	**32.89**	**0.9573**	**0.2001**	1674.0
11	*32.88*	0.9570	0.2039	1677.2
12	**32.89**	*0.9571*	*0.2022*	1681.4

In comparison with existing methods, HACR-Net exhibits a consistently favorable efficiency–performance trade-off. For instance, although VDSR [19] employs fewer parameters (665k), it incurs a substantially higher computational burden (1,225.2G FLOPs), whereas RCAN [36] significantly increases parameter count (12,460k) to achieve lower FLOPs (24.5G). Similarly, despite having a comparable parameter scale to MFER [27], HACR-Net reduces computational cost and achieves faster inference (1.25s vs. 1.92s), also outperforming CSN [14] and SwinIR [50] in runtime. HACR-Net design is between intermediate and ultralightweight architectures such as SRCNN [18] and large-scale networks such as RDN [20], while maintaining reasonable computational efficiency. More notably, compared with the transformer-based Restormer [51], HACR-Net achieves comparable FLOPs with fewer parameters.

### Ablation study

We conduct a series of ablation experiments to evaluate the impact of key components of HACR-Net, including HAM, the number of CAAMs, and its two blocks (MFAB and CRAB).

#### Ablation of the number of CAAM.

To examine the trade-off between reconstruction quality and computational efficiency, and to assess the impact of CAAM quantity, we conducted an ablation study by varying the number of CAAM modules. As shown in Table 4, performance consistently improves as the number of CAAMs increases, but the gains in PSNR, SSIM, and LPIPS become negligible beyond 10 modules. During this increase, the parameter count rises only slightly from 1.67M at 7 CAAMs to 1.68M at 12 CAAMs, which reflects a clear balance between quality and complexity. Using 10 CAAMs provides the optimal configuration, yielding the highest PSNR and SSIM with a lower LPIPS while maintaining a moderate parameter count. Additional stacking leads to diminishing returns with increased computational overhead, whereas reducing to 7 CAAMs minimally lowers parameters but results in a measurable 0.28 dB drop in PSNR. Overall, the ablation confirms that HACR‑Net is structured to maximize reconstruction fidelity while retaining practical efficiency.

Our architectural choices were guided by systematic ablation studies that explicitly balance reconstruction fidelity and computational efficiency. Convolutional kernel sizes across HACR-Net were selected based on their optimal performance–parameter trade-off, achieving strong reconstruction quality without unnecessary model complexity. The placement of the HAM in the shallow layers prior to deep feature extraction was empirically validated through ablation experiments, yielding improvement over alternative placements. This indicates that early attention-based filtering suppresses irrelevant features before they propagate through subsequent CAAMs. The 7×7 kernel used in the spatial attention branch was chosen to effectively capture broader anatomical context while maintaining computational efficiency. The number of CAAMs (10 modules) and the bottleneck width of the CRAB were determined based on the performance saturation trends observed in Tables 4 and 5, beyond which further architectural complexity resulted in diminishing gains. Collectively, these design decisions enable HACR-Net to achieve state-of-the-art reconstruction performance with only 1.67M parameters and 81.3G FLOPs.

**Table 5:** Ablation on CRAB with and without a bottleneck design on IXI (PD and T1) ×2 and ×4 scaling factor.

		×2			×4		
		PSNR↑	SSIM↑	LPIPS↓	PSNR↑	SSIM↑	LPIPS↓
PD	CRAB/w	42.10	0.9907	0.0835	33.88	0.9583	0.1786
	CRAB/wo	41.79	0.9895	0.0963	33.75	0.9544	0.2017
T1	CRAB/w	39.34	0.9867	0.1044	32.71	0.9308	0.2014
	CRAB/wo	38.66	0.9824	0.1180	32.16	0.9245	0.2221

CRAB/w = CRAB with bottleneck, CRAB/wo = CRAB without bottleneck.

#### Ablation study on CRAB with and without a bottleneck.

To evaluate the effectiveness of the CRAB module, we performed an ablation study comparing variants with and without the bottleneck design. As shown in Table 5, incorporating the bottleneck consistently improves reconstruction quality across the modalities at ×2 and ×4 scaling factors. For example, in the PD case at ×2, the PSNR increased from 41.79 dB to 42.10 dB and the LPIPS decreased from 0.0963 to 0.0835. Meanwhile, at ×4, the SSIM improved from 0.9544 to 0.9583. Similarly, in the T1 modality, the bottleneck yielded a gain of nearly 0.7dB in PSNR (from 38.66 to 39.34 dB) at ×2 and reduced LPIPS from 0.2221 to 0.2014 at ×4. These results confirm that the bottleneck design, which maintains a wide channel width, effectively mitigates information loss during feature reduction and facilitates the recovery of fine contextual details.

#### Ablation study on the effectiveness of HAM, MFAB, and CRAB.

We conducted an extensive ablation study to assess the individual and joint contributions of the three core modules, HAM, MFAB, and CRAB, to the overall performance of HACR-Net. The experiments were performed on the IXI (PD) dataset at a ×2 up-sampling factor as summarized in Table 6. The baseline model with all three modules removed achieves a PSNR of 40.50 dB. When the modules are added individually, MFAB and CRAB provide incremental gains (40.62 dB and 40.69 dB, respectively), with CRAB offering the largest uplift (+0.19 dB), validating its effectiveness in channel retention and deep-feature refinement.

**Table 6:** Ablation study results for HAM, MFAB, and CRAB blocks on the IXI PD dataset at ×2 magnification.

Ablation Settings			Parameters	FLOPs	IXI (PD)
HAM	MFAB	CRAB			PSNR/SSIM/LPIPS
✓	×	×	1468k	80.0G	40.50/0.9839/0.0994
×	✓	×	1471k	80.0G	40.62/0.9846/0.0983
×	×	✓	1466k	80.5G	40.69/0.9851/0.0978
×	✓	✓	1652k	81.0G	41.82/0.9897/0.0847
✓	✓	×	1632k	81.0G	41.73/0.9885/0.0851
✓	✓	✓	1674k	81.3G	42.10/0.9907/0.0835

Beyond the individual performance gains, a clear synergistic effect is observed among the proposed modules. Pairwise combinations such as HAM+MFAB (41.82 dB) and HAM+CRAB (41.73 dB), show substantial enhancements over single-module variants, indicating complementary roles. HAM facilitates shallow-feature preservation, MFAB enhances multi-scale feature aggregation, and CRAB reinforces deep-feature retention. Integrating all three components yields the best performance, with the full model achieving 42.10 dB/0.9907/0.0835 (PSNR/SSIM/LPIPS). This represents a 0.28–0.37 dB improvement over any two-module configuration, demonstrating that the modules are not redundant but instead work collaboratively to optimize the feature space.

We further examine the trade-off between model complexity and performance across individual modules and their combined configuration, as summarized in Table 6. Although incorporating HAM, MFAB, and CRAB increases the parameter count and computational cost, their combination yields consistent improvements in PSNR, SSIM, and LPIPS. These results demonstrate that the additional complexity is justified by the corresponding quantitative gains.

To ensure statistical rigor, Table 7 reports the mean and standard deviation of PSNR and SSIM for the PD, T1, and T2 modalities, computed over 11,136 test images. The standard deviation was calculated across all individual test slices to reflect subject-level variability. These results offer a more comprehensive view of the model’s consistency and robustness across the full benchmark dataset.

**Table 7:** Mean and standard deviation of quantitative metrics on the PD, T1 and T2 test set.

Dataset	Scaling Factor	PSNR Mean ± SD	SSIM Mean ± SD
PD	×2	42.10±1.201	0.9907±0.0066
	×4	33.88±1.425	0.9583±0.0087
T1	×2	39.34±1.186	0.9867±0.0055
	×4	32.71±1.465	0.9308±0.0085
T2	×2	41.15±1.117	0.9891±0.0163
	×4	32.89±1.328	0.9573±0.0159

## Conclusion

In this work, we present HACR-Net, a model designed to address key challenges in brain MRI SR. The framework effectively preserves structural boundaries, tissue interfaces, and multiscale features while maintaining a compact and computationally efficient architecture. The integration of HAM strengthens shallow feature extraction by prioritizing informative channels and spatially significant regions. At the same time, the CAAM module, comprising the MFAB and CRAB blocks, synergistically enhances reconstruction fidelity by capturing fine-grained details and preserving contextual information with minimal feature loss. Evaluations on the IXI and BraTS 2018 benchmark datasets confirm that HACR-Net achieves consistent improvements over state-of-the-art methods at scaling factors of ×2 and ×4. By combining competitive reconstruction accuracy with substantially reduced computational cost, HACR-Net demonstrates a balanced advancement in both performance and efficiency.

## Future work

Future extensions of HACR-Net will focus on improving anatomical fidelity, enhancing efficiency, and advancing clinical applicability. Feature representation across spatial hierarchies will be strengthened by incorporating multi-scale attention-guided fusion strategies, while edge-aware loss functions such as gradient consistency and structural dissimilarity will be explored to improve boundary precision and high-frequency detail reconstruction. To address the computational demands of real-time clinical deployment, we will investigate architectural optimizations including depthwise separable convolutions, group convolutions, structured pruning, and quantization-aware training, with the goal of reducing inference time and model size without compromising image quality. To help bridge the gap between synthetic LR data and real clinical conditions, future work will broaden the evaluation by incorporating additional MRI degradation scenarios, such as k-space undersampling or anisotropic resolution, enabling future studies to extend the evaluation to a wider range of clinically relevant conditions.

## References

[1] Hussain S, Mubeen I, Ullah N, Shah SSUD, Khan BA, Zahoor M, et al. Modern Diagnostic Imaging Technique Applications and Risk Factors in the Medical Field: A Review. Biomed Res Int. 2022;2022:5164970. doi: 10.1155/2022/5164970 35707373 PMC9192206

[2] Qiu D, Zhang S, Liu Y, Zhu J, Zheng L. Super-resolution reconstruction of knee magnetic resonance imaging based on deep learning. Comput Methods Programs Biomed. 2020;187:105059. doi: 10.1016/j.cmpb.2019.105059 31582263

[3] Ren J, Li J, Chen S, Liu Y, Ta D. Unveiling the potential of ultrasound in brain imaging: Innovations, challenges, and prospects. Ultrasonics. 2025;145:107465. doi: 10.1016/j.ultras.2024.107465 39305556

[4] Kennedy JA, Israel O, Frenkel A, Bar-Shalom R, Azhari H. Super-resolution in PET imaging. IEEE Trans Med Imaging. 2006;25(2):137–47. doi: 10.1109/TMI.2005.861705 16468448

[5] Tsapaki V. Radiation dose optimization in diagnostic and interventional radiology: Current issues and future perspectives. Phys Med. 2020;79:16–21. doi: 10.1016/j.ejmp.2020.09.015 33035737

[6] Koutsiaris AG, Batis V, Liakopoulou G, Tachmitzi SV, Detorakis ET, Tsironi EE. Optical Coherence Tomography Angiography (OCTA) of the eye: A review on basic principles, advantages, disadvantages and device specifications. Clin Hemorheol Microcirc. 2023;83(3):247–71. doi: 10.3233/CH-221634 36502308

[7] Xu L, Li G, Chen Q. Accurate and lightweight MRI super-resolution via multi-scale bidirectional fusion attention network. PLoS One. 2022;17(12):e0277862. doi: 10.1371/journal.pone.0277862 36520931 PMC9754592

[8] Pfaehler E, Pflugfelder D, Scharr H. Untrained perceptual loss for image denoising of line-like structures in MR images. PLoS One. 2025;20(2):e0318992. doi: 10.1371/journal.pone.0318992 40009630 PMC11864525

[9] Plenge E, Poot DHJ, Bernsen M, Kotek G, Houston G, Wielopolski P, et al. Super-resolution methods in MRI: can they improve the trade-off between resolution, signal-to-noise ratio, and acquisition time?. Magn Reson Med. 2012;68(6):1983–93. doi: 10.1002/mrm.24187 22298247

[10] Muhammad A, Aramvith S, Duangchaemkarn K, Sun M-T. Brain MRI Image Super-Resolution Reconstruction: A Systematic Review. IEEE Access. 2024;12:156347–62. doi: 10.1109/access.2024.3478829

[11] Fang C, Zhang D, Wang L, Zhang Y, Cheng L, Han J. Cross-Modality High-Frequency Transformer for MR Image Super-Resolution. In: Proceedings of the 30th ACM International Conference on Multimedia, 2022. 1584–92. 10.1145/3503161.3547804

[12] You S, Lei B, Wang S, Chui CK, Cheung AC, Liu Y, et al. Fine Perceptive GANs for Brain MR Image Super-Resolution in Wavelet Domain. IEEE Trans Neural Netw Learn Syst. 2023;34(11):8802–14. doi: 10.1109/TNNLS.2022.3153088 35254996

[13] Chen J, Wu F, Wang W. Joint MR image reconstruction and super-resolution via mutual co-attention network. Journal of Computational Design and Engineering. 2023;11(1):288–304. doi: 10.1093/jcde/qwae006

[14] Zhao X, Zhang Y, Zhang T, Zou X. Channel Splitting Network for Single MR Image Super-Resolution. IEEE Trans Image Process. 2019;28(11):5649–62. doi: 10.1109/TIP.2019.2921882 31217110

[15] Hajian A, Aramvith S. AERU-Net: Adaptive Edge Recovery and Attention U-Shaped Network for Remote Sensing Image Super-Resolution. IEEE Access. 2025;13:59177–97. doi: 10.1109/access.2025.3556312

[16] Luo X, Ai Z, Liang Q, Liu D, Xie Y, Qu Y, et al. AdaFormer: Efficient Transformer with Adaptive Token Sparsification for Image Super-resolution. AAAI. 2024;38(5):4009–16. doi: 10.1609/aaai.v38i5.28194

[17] Wang S, Liu J, Wan B, Li W. Hybrid feature fusion neural network integrating transformer for DCE-MRI super resolution. Biomedical Signal Processing and Control. 2023;86:105342. doi: 10.1016/j.bspc.2023.105342 PMC10083211 37082352

[18] Dong C, Loy CC, He K, Tang X. Image Super-Resolution Using Deep Convolutional Networks. IEEE Trans Pattern Anal Mach Intell. 2016;38(2):295–307. doi: 10.1109/TPAMI.2015.2439281 26761735

[19] Kim J, Lee JK, Lee KM. Accurate Image Super-Resolution Using Very Deep Convolutional Networks. In: 2016 IEEE Conference on Computer Vision and Pattern Recognition (CVPR), 2016. 1646–54. 10.1109/cvpr.2016.182

[20] Zhang Y, Tian Y, Kong Y, Zhong B, Fu Y. Residual Dense Network for Image Super-Resolution. In: 2018 IEEE/CVF Conference on Computer Vision and Pattern Recognition, 2018. 2472–81. 10.1109/cvpr.2018.00262

[21] Li J, Fang F, Mei K, Zhang G. Multi-scale Residual Network for Image Super-Resolution. Lecture Notes in Computer Science. Springer International Publishing. 2018. p. 527–42. 10.1007/978-3-030-01237-3_32

[22] Feng CM, Fu H, Yuan S, Xu Y. Multi-contrast MRI super-resolution via a multi-stage integration network. In: International Conference on Medical Image Computing and Computer-Assisted Intervention, 2021. 140–9.

[23] Weng X, Chen Y, Zheng Z, Gu Y, Zhou J, Zhang Y. A high-frequency focused network for lightweight single image super-resolution. 2023. https://arxiv.org/abs/2303.11701

[24] Yang Y, Qi Y. Hierarchical accumulation network with grid attention for image super-resolution. Knowledge-Based Systems. 2021;233:107520. doi: 10.1016/j.knosys.2021.107520

[25] Zou B, Ji Z, Zhu C, Dai Y, Zhang W, Kui X. Multi-scale deformable transformer for multi-contrast knee MRI super-resolution. Biomedical Signal Processing and Control. 2023;79:104154. doi: 10.1016/j.bspc.2022.104154

[26] Wang H, Hu X, Zhao X, Zhang Y. Wide Weighted Attention Multi-Scale Network for Accurate MR Image Super-Resolution. IEEE Trans Circuits Syst Video Technol. 2022;32(3):962–75. doi: 10.1109/tcsvt.2021.3070489

[27] Li H, Jia Y, Zhu H, Han B, Du J, Liu Y. Multi-level feature extraction and reconstruction for 3D MRI image super-resolution. Comput Biol Med. 2024;171:108151. doi: 10.1016/j.compbiomed.2024.108151 38387383

[28] Puttagunta M, Subban R, Babu C NK. SwinIR Transformer Applied for Medical Image Super-Resolution. Procedia Computer Science. 2022;204:907–13. doi: 10.1016/j.procs.2022.08.110 PMC9464299 36120410

[29] Chen K, Li L, Liu H, Li Y, Tang C, Chen J. SwinFSR: Stereo Image Super-Resolution using SwinIR and Frequency Domain Knowledge. In: 2023 IEEE/CVF Conference on Computer Vision and Pattern Recognition Workshops (CVPRW), 2023. 1764–74. 10.1109/cvprw59228.2023.00177

[30] Ikebe Y, Fujima N, Kameda H, Harada T, Shimizu Y, Kwon J, et al. Ultra-fast whole-brain T2-weighted imaging in 7 seconds using dual-type deep learning reconstruction with single-shot acquisition: clinical feasibility and comparison with conventional methods. Jpn J Radiol. 2026;44(1):35–42. doi: 10.1007/s11604-025-01875-6 41003971 PMC12769672

[31] Sherif FM, Elmogy SA, Denewar FA. Utility of deep learning reconstruction to reach the magic triangle in brain MRI. Egypt J Radiol Nucl Med. 2025;56(1). doi: 10.1186/s43055-025-01574-2

[32] Liu X, Huang C, Meng J, Chen Q, Ji W, Wang Q. Super-Resolution Reconstruction Approach for MRI Images Based on Transformer Network. AI. 2025;6(11):291. doi: 10.3390/ai6110291

[33] Anwar S, Barnes N. Densely Residual Laplacian Super-Resolution. IEEE Trans Pattern Anal Mach Intell. 2022;44(3):1192–204. doi: 10.1109/TPAMI.2020.3021088 32877331

[34] Suryanarayana G, Nimmagadda SM, Nageswara Rao S, Y Mahnashi AM, Kondamuri SR, Hussain Ahmadini AA, et al. Enhanced MRI-PET fusion using Laplacian pyramid and empirical mode decomposition for improved oncology imaging. PLoS One. 2025;20(5):e0322443. doi: 10.1371/journal.pone.0322443 40388483 PMC12088072

[35] Hu J, Shen L, Sun G. Squeeze-and-Excitation Networks. In: 2018 IEEE/CVF Conference on Computer Vision and Pattern Recognition, 2018. 7132–41. 10.1109/cvpr.2018.00745

[36] Zhang Y, Li K, Li K, Wang L, Zhong B, Fu Y. Image Super-Resolution Using Very Deep Residual Channel Attention Networks. Lecture Notes in Computer Science. Springer International Publishing. 2018. p. 294–310. 10.1007/978-3-030-01234-2_18

[37] Kim J, Lee JK, Lee KM. Deeply-Recursive Convolutional Network for Image Super-Resolution. In: 2016 IEEE Conference on Computer Vision and Pattern Recognition (CVPR), 2016. 1637–45. 10.1109/cvpr.2016.181

[38] Tai Y, Yang J, Liu X. Image Super-Resolution via Deep Recursive Residual Network. In: 2017 IEEE Conference on Computer Vision and Pattern Recognition (CVPR), 2017. 2790–8. 10.1109/cvpr.2017.298

[39] Wang C, Li Z, Shi J. Lightweight image super-resolution with adaptive weighted learning network. arXiv preprint arXiv:190402358. 2019.

[40] Dai T, Cai J, Zhang Y, Xia S-T, Zhang L. Second-Order Attention Network for Single Image Super-Resolution. In: 2019 IEEE/CVF Conference on Computer Vision and Pattern Recognition (CVPR), 2019. 11057–66. 10.1109/cvpr.2019.01132

[41] Tian C, Xu Y, Zuo W, Zhang B, Fei L, Lin C-W. Coarse-to-Fine CNN for Image Super-Resolution. IEEE Trans Multimedia. 2021;23:1489–502. doi: 10.1109/tmm.2020.2999182

[42] Fang F, Li J, Zeng T. Soft-edge Assisted Network for Single Image Super-Resolution. IEEE Trans Image Process. 2020. doi: 10.1109/TIP.2020.2973769 32092001

[43] Sun L, Liu Z, Sun X, Liu L, Lan R, Luo X. Lightweight Image Super-Resolution via Weighted Multi-Scale Residual Network. IEEE/CAA J Autom Sinica. 2021;8(7):1271–80. doi: 10.1109/jas.2021.1004009

[44] Sun L, Pan J, Tang J. Shufflemixer: An efficient convnet for image super-resolution. Advances in Neural Information Processing Systems. 2022;35:17314–26.

[45] Woo S, Park J, Lee J-Y, Kweon IS. CBAM: Convolutional Block Attention Module. Lecture Notes in Computer Science. Springer International Publishing. 2018. p. 3–19. 10.1007/978-3-030-01234-2_1

[46] Itti L, Koch C, Niebur E. A model of saliency-based visual attention for rapid scene analysis. IEEE Trans Pattern Anal Machine Intell. 1998;20(11):1254–9. doi: 10.1109/34.730558

[47] -Liu J, Zhang W, Tang Y, Tang J, Wu G. Residual Feature Aggregation Network for Image Super-Resolution. In: 2020 IEEE/CVF Conference on Computer Vision and Pattern Recognition (CVPR), 2020. 2356–65. 10.1109/cvpr42600.2020.00243

[48] Wang Q, Wu B, Zhu P, Li P, Zuo W, Hu Q. ECA-Net: Efficient channel attention for deep convolutional neural networks. In: Proceedings of the IEEE/CVF conference on computer vision and pattern recognition; 2020. p. 11534–42.

[49] Zhang Y, Li K, Li K, Fu Y. MR Image Super-Resolution with Squeeze and Excitation Reasoning Attention Network. In: 2021 IEEE/CVF Conference on Computer Vision and Pattern Recognition (CVPR), 2021. 13420–9. 10.1109/cvpr46437.2021.01322

[50] Liang J, Cao J, Sun G, Zhang K, Van Gool L, Timofte R. Swinir: Image restoration using swin transformer. In: Proceedings of the IEEE/CVF international conference on computer vision; 2021. p. 1833-–44.

[51] Zamir SW, Arora A, Khan S, Hayat M, Khan FS, Yang MH. Restormer: Efficient transformer for high-resolution image restoration. In: Proceedings of the IEEE/CVF conference on computer vision and pattern recognition, 2022. 5728–39.

[52] Wang W, Shen H, Chen J, Xing F. MHAN: Multi-Stage Hybrid Attention Network for MRI reconstruction and super-resolution. Comput Biol Med. 2023;163:107181. doi: 10.1016/j.compbiomed.2023.107181 37352637

[53] Hua X, Du Z, Ma J, Yu H. Multi kernel cross sparse graph attention convolutional neural network for brain magnetic resonance imaging super-resolution. Biomedical Signal Processing and Control. 2024;96:106444. doi: 10.1016/j.bspc.2024.106444

[54] He C, Liu H, Shen Y, Zhou D, Wu L, Ma H, et al. Improving the magnetic resonance images super-resolution with a dual-channel enhancement model incorporating complementary information. Engineering Applications of Artificial Intelligence. 2025;148:110359. doi: 10.1016/j.engappai.2025.110359

[55] Rasheed Z, Ma Y-K, Ullah I, Al-Khasawneh M, Almutairi SS, Abohashrh M. Integrating Convolutional Neural Networks with Attention Mechanisms for Magnetic Resonance Imaging-Based Classification of Brain Tumors. Bioengineering (Basel). 2024;11(7):701. doi: 10.3390/bioengineering11070701 39061782 PMC11273980

[56] Rasheed Z, Ma YK, Bharany S, Shandilya G, Ullah I, Ali F. Classification of MRI brain tumor with hybrid VGG19 and ensemble classifier approach. In: 2024 First International Conference on Innovations in Communications, Electrical and Computer Engineering (ICICEC), 2024. 1–7.

[57] Ahmad I, Liu Z, Li L, Ullah I, Aboyeji ST, Wang X, et al. Robust Epileptic Seizure Detection Based on Biomedical Signals Using an Advanced Multi-View Deep Feature Learning Approach. IEEE J Biomed Health Inform. 2024;28(10):5742–54. doi: 10.1109/JBHI.2024.3396130 38696293

[58] Ahmad I, Zhu M, Liu Z, Shabaz M, Ullah I, Tong MCF, et al. Multi-Feature Fusion-Based Convolutional Neural Networks for EEG Epileptic Seizure Prediction in Consumer Internet of Things. IEEE Trans Consumer Electron. 2024;70(3):5631–43. doi: 10.1109/tce.2024.3363166

[59] Ahmed MJ, Afridi U, Shah HA, Khan H, Bhatt MW, Alwabli A, et al. CardioGuard: AI-driven ECG authentication hybrid neural network for predictive health monitoring in telehealth systems. SLAS Technol. 2024;29(5):100193. doi: 10.1016/j.slast.2024.100193 39307457

[60] Rahman AU, Ali S, Wason R, Aggarwal S, Abohashrh M, Daradkeh YI, et al. Emotion‐Based Mental State Classification Using EEG for Brain‐Computer Interface Applications. Computational Intelligence. 2025;41(4). doi: 10.1111/coin.70112

[61] He K, Zhang X, Ren S, Sun J. Deep residual learning for image recognition. In: Proceedings of the IEEE conference on computer vision and pattern recognition; 2016. p. 770-–8.

[62] Johnson J, Alahi A, Fei-Fei L. Perceptual Losses for Real-Time Style Transfer and Super-Resolution. Lecture Notes in Computer Science. Springer International Publishing. 2016. p. 694–711. 10.1007/978-3-319-46475-6_43

[63] Charbonnier P, Blanc-Feraud L, Aubert G, Barlaud M. Two deterministic half-quadratic regularization algorithms for computed imaging. In: Proceedings of 1st International Conference on Image Processing. 168–72. 10.1109/icip.1994.413553

[64] Goodfellow IJ, Pouget-Abadie J, Mirza M, Xu B, Warde-Farley D, Ozair S. Generative adversarial nets. Advances in Neural Information Processing Systems. 2014;27.

[65] Menze BH, Jakab A, Bauer S, Kalpathy-Cramer J, Farahani K, Kirby J, et al. The Multimodal Brain Tumor Image Segmentation Benchmark (BRATS). IEEE Trans Med Imaging. 2015;34(10):1993–2024. doi: 10.1109/TMI.2014.2377694 25494501 PMC4833122

[66] Bakas S, Akbari H, Sotiras A, Bilello M, Rozycki M, Kirby JS, et al. Advancing The Cancer Genome Atlas glioma MRI collections with expert segmentation labels and radiomic features. Sci Data. 2017;4:170117. doi: 10.1038/sdata.2017.117 28872634 PMC5685212

[67] Shi F, Cheng J, Wang L, Yap P-T, Shen D. LRTV: MR Image Super-Resolution With Low-Rank and Total Variation Regularizations. IEEE Trans Med Imaging. 2015;34(12):2459–66. doi: 10.1109/TMI.2015.2437894 26641727 PMC5572670

[68] Shi J, Li Z, Ying S, Wang C, Liu Q, Zhang Q, et al. MR Image Super-Resolution via Wide Residual Networks With Fixed Skip Connection. IEEE J Biomed Health Inform. 2019;23(3):1129–40. doi: 10.1109/JBHI.2018.2843819 29993565

[69] Kingma DP. Adam: A method for stochastic optimization. arXiv preprint arXiv:14126980. 2014.

[70] Wang Z, Bovik AC, Sheikh HR, Simoncelli EP. Image quality assessment: from error visibility to structural similarity. IEEE Trans Image Process. 2004;13(4):600–12. doi: 10.1109/tip.2003.819861 15376593

[71] Zhang R, Isola P, Efros AA, Shechtman E, Wang O. The Unreasonable Effectiveness of Deep Features as a Perceptual Metric. In: 2018 IEEE/CVF Conference on Computer Vision and Pattern Recognition, 2018. 586–95. 10.1109/cvpr.2018.00068

[72] Ma C, Yang C-Y, Yang X, Yang M-H. Learning a no-reference quality metric for single-image super-resolution. Computer Vision and Image Understanding. 2017;158:1–16. doi: 10.1016/j.cviu.2016.12.009

[73] Lim B, Son S, Kim H, Nah S, Mu Lee K. In: Proceedings of the IEEE Conference on Computer Vision and Pattern Recognition Workshops, 2017. 136–44.

